# Comparison of anticholinergic burden with chronic polypharmacy on functional decline and mortality in Korean older people: a retrospective nationwide cohort study

**DOI:** 10.1186/s12877-024-04692-0

**Published:** 2024-01-23

**Authors:** Youn Huh, Ye-Jee Kim, Jung-Yeon Choi, Ji Eun Lee, Hee-Won Jung, Cheol Min Shin, Chang Won Won, Ki Young Son

**Affiliations:** 1 Department of Family Medicine, Uijeongbu Eulji Medical Center, Eulji University, Gyeonggi-do, Republic of Korea; 2 Department of Clinical Epidemiology and Biostatistics, Asan Medical Center, University of Ulsan College of Medicine, Seoul, Republic of Korea; 3 Department of Internal Medicine, Seoul National University Bundang Hospital, Gyeonggi-do, Republic of Korea; 4 Department of Family Medicine, Seoul National University Hospital, Seoul, Republic of Korea; 5 Division of Geriatrics, Department of Internal Medicine, Asan Medical Center, University of Ulsan College of Medicine, Seoul, Republic of Korea; 6 Department of Family Medicine, College of Medicine, Kyung Hee University, Seoul, Republic of Korea; 7 Department of Family Medicine, Asan Medical Center, University of Ulsan College of Medicine, 88 Olympic-ro 43 gil, Songpa-gu, 05505 Seoul, Korea

**Keywords:** Polypharmacy, Anticholinergic burden, Functional decline, All-cause mortality, Korean, Older people

## Abstract

**Background:**

We aimed to evaluate the association of anticholinergic burden and chronic polypharmacy with the incidence of functional decline and all-cause mortality, and to determine the difference between anticholinergic burden and chronic polypharmacy among Korean older people.

**Methods:**

This nationwide cohort study included 42,132 older people aged ≥ 65 years who underwent Korean National Health Insurance Service health examinations from 2007 to 2008. Odds ratios (ORs) and 95% confidence intervals (CIs) for abnormal Timed Up and Go (TUG) test results were assessed using multivariate logistic regression analyses. Hazard ratios (HRs) and 95% CIs for all-cause mortality until the end of 2015 were estimated using multivariable Cox proportional hazards regression analysis.

**Results:**

Of the participants, 37.19% had abnormal TUG test results, and 7.66% of those died during the 5.7-year mean follow-up. The abnormal TUG test results OR increased by 27% among individuals with Korean Anticholinergic Burden Scale (KABS) scores ≥ 3 (OR 1.27, 95% CI 1.02–1.58) compared to those with KABS scores of 0. The HRs for all-cause mortality increased for individuals with higher KABS scores (P for trend < 0.001) or chronic polypharmacy (P for trend < 0.001) compared to those for individuals without these conditions. The combination of a higher KABS or chronic polypharmacy and abnormal TUG test results increased the risk of all-cause mortality (All *P* for trend < 0.001).

**Conclusion:**

Anticholinergic drug burden shows a better association with functional decline than chronic polypharmacy, and the use of medications and functional decline may be important risk factors for all-cause mortality among older people.

**Supplementary Information:**

The online version contains supplementary material available at 10.1186/s12877-024-04692-0.

## Background

The number and prevalence of older people aged ≥ 60 years is increasing worldwide, from 1 billion (12%) in 2020 to 2.1 billion (22%) by 2050. In particular, the number of older people aged ≥ 80 years will increase by more than three times in the same period [1]. In the United States (US), the population of older people ≥ 65 years was 16% of the population in 2019 and will increase to 22% by 2040. In Korea, the proportion of the older people ≥ 65 years has been increasing rapidly, from 7% (an aging society) in 2000 to 14% (an aged society) in 2017, and is expected to reach 21% (a super-aging society) in 2026 [2].

The mean number of chronic diseases in older people is 3.0 for men and 4.5 for women, [3]. Therefore, the number of medications and the risk of side effects is also increased [4]. Polypharmacy, defined as taking ≥ 5 medications, is present in approximately 23% of older people [5] and is associated with potentially inappropriate medications (PIMs) [6], falls, functional decline [7], and mortality [8]. Anticholinergic drug burden is the cumulative effect of various medications as PIMs and is associated with geriatric syndromes, such as cognitive decline, confusion, falls, and physical decline [9]. In addition, it was often overlooked by physicians, but might increase the risks of mortality, hospitalization, and length of hospital stay among older people [10].

Frailty is a state of increased vulnerability due to the loss of the capacity to maintain homeostasis, resulting in the decreased function of multiple organ systems and an increased risk of disability, falls, hospitalization, and all-cause mortality in older people [11]. Previous studies have shown that polypharmacy is associated with an increased risk of frailty in older people [12]. Frailty consists of medical use, self-reported health status, polypharmacy, weight loss, depressive mood, incontinence, visual or auditory problems, and functional decline [13]. Because physical function decline is the cornerstone of physical frailty in clinical situation, the present study is focused on physical function decline measured by Timed Up and Go (TUG) test.

Some studies have shown that polypharmacy and anticholinergic drug burden increase the risk of mortality [8, 14]. However, few studies have evaluated representative nationwide data, and there is limited evidence of an association of chronic polypharmacy and anticholinergic drug burden with functional decline and all-cause mortality in older people. Therefore, this study evaluated these associations and to determine the difference between anticholinergic burden and chronic polypharmacy among older people using nationwide cohort data from the Korea National Health Insurance Service-Senior (NHIS-Senior) database.

## Methods

### Data Source and Study Population

This study retrospectively evaluated nationwide cohort data from the NHIS-Senior database. The NHIS-Senior database consisted of data from 558,147 older people aged ≥ 60 years (10% of 5.5 million total older people) who represented older people residing in Korea and were randomly selected. These data were used to implement health policies and research. The NHIS is the only payer under Korea’s single-insurer nationwide health insurance system. Most Korean citizens are enrolled in the NHIS and classified as insured, self-employed, and medical benefit recipients. Therefore, the NHIS retains an extensive medical database of nearly the entire South Korean population regarding demographic characteristics, health examinations, disease diagnoses, medical treatments, health screenings, and procedures based on medical claims according to the International Classification of Diseases 10th Revision (ICD-10) codes. In addition, the health screening database consists of the National Screening Program for Transitional Ages, which is conducted for patients aged 40 or 66 years [15]. Since 2015, NHIS data have been made accessible to qualified researchers who have submitted study protocols approved by official review committees.

A total of 558,147 individuals were identified from the database. We excluded individuals who did not participate in the National Screening Program for Transitional Ages with an age of 66 during 2007–2008 because they did not include the TUG test (*n* = 1,352) and those with missing data for important variables of the study such as physical activity (*n* = 514,663). Finally, 42,132 individuals (19,737 men and 22,395 women) eligible for analyses were followed-up until December 31, 2017. This study adhered to the principles of the Declaration of Helsinki and was approved by the Institutional Review Board of Kyung Hee University Medical Center (KHUH 2020-05-051). The requirement for informed consent was waived because we used anonymized data from the participants.

### Study outcomes

The outcomes of this study were functional decline and all-cause mortality. Functional decline was defined by assessing physical frailty using the TUG test included in the National Screening Program for Transitional Ages for participants of 66 years. A TUG test result of < 10 s in the year of the health examination was defined as normal, and the opposite was defined as abnormal [16]. All-cause mortality was defined as having a date of death between January 1, 2007 and December 31, 2015.

### Definitions of anticholinergic drug burden and chronic polypharmacy

Anticholinergic drug burden was evaluated using the Korean Anticholinergic Burden Scale (KABS) and was classified using scores of 0, 1–2, or ≥ 3 [17]. The KABS, developed as a Korean-specific anticholinergic burden scale, was created through a literature review and a modified Delphi process [17, 18]. Supplementary Table S1 shows medication lists according to the Korean Anticholinergic Burden Scale including this study. However, specific drug details are unavailable. Chronic polypharmacy was defined as taking 5 or more medications. Medications were included in analysis only when taken for ≥ 250 days during the year preceding the health examination.

### Covariates

The NHIS-Senior database contains detailed information regarding demographic characteristics and lifestyle, which were evaluated using standardized self-administered questionnaires. The lowest 5% of the income range of the participants was classified as low income, while the remaining was considered non-low income. The smoking status was classified into three categories: current smokers, defined as those who smoked more than 100 cigarettes in their lifetime and currently smoke; ex-smokers, defined as those who smoked more than 100 cigarettes in their lifetime but did not currently smoke; and never smokers, defined as those who smoked less than 100 cigarettes in their lifetime. Risky alcohol drinkers were defined as participants who consumed more than 3 drinks of alcohol at a time. Regular exercise was defined as high-intensity exercise for ≥ 3 times per week or moderate-intensity exercise for ≥ 5 times per week. A depressive mood was defined by “yes” to at least one of the 3 questions of Korean Version of Geriatric Depression Scale during the past week. Cognitive impairment was defined by a score of ≥ 6 obtained by assigning three points to each of the 15 cognitive function questions during the previous year. Falls were defined as having a fall within 6 months. An impairment of activities of daily living was defined as answering “no” to three of six questions.

Health examinations, including anthropometric and laboratory measurements, were conducted by qualified medical staff. The height, weight, and waist circumference were measured, and the body mass index (BMI) was calculated as the weight divided by the square of the height. Systolic and diastolic blood pressures were measured while participants were seated after 5 min of rest. Blood samples were obtained after overnight fasting to determine serum concentrations of glucose and total cholesterol.

Comorbidities were identified based on the health examination results, medical claims for disease diagnoses, and medication prescriptions. Hypertension was defined as having a blood pressure of ≥ 140/90 mmHg or at least one medication prescription claim for ≥ 250 days in the year prior to the health examination with ICD-10 codes I10–I13 or I15. Diabetes was defined as having a fasting plasma glucose of ≥ 126 mg/dL or at least one medication or insulin prescription claim for ≥ 250 days in the year prior to the health examination with ICD-10 codes E11–E14. Dyslipidemia was defined as having a total cholesterol concentration of ≥ 240 mg/dL or at least one medication prescription claim for ≥ 250 days in the year prior to the health examination with ICD-10 code E78.

### Statistical analyses

All statistical analyses were performed using STATA ware (version 16.1; STATA. Corp, College Station, Texas). The baseline characteristics are shown as means ± standard deviations for continuous variables or numbers (percentages) for categorical variables. Continuous variables were compared using the independent t-test, and categorical variables were compared using the chi-square test. We performed a multivariate logistic regression analysis and calculated odds ratios (ORs) and 95% confidence intervals (CIs) to determine the association between abnormal TUG test results and the KABS score and chronic polypharmacy. In addition to the crude model, three models were used: model 1 was adjusted for sex, BMI, and income; model 2 was additionally adjusted for smoking status, alcohol consumption, and physical activity; and model 3 was additionally adjusted for depressive mood, cognitive impairment, activities of daily living, and falls. The incidence of all-cause mortality was calculated by dividing the number of events by 1000 person-years. We performed a multivariate Cox proportional hazards regression analysis to evaluate the association of the KABS score and chronic polypharmacy with the risk of all-cause mortality. The results were presented as hazard ratios (HRs) and 95% CIs. In addition to the crude model, two models were used: model 1 was adjusted for sex, BMI, income, smoking status, alcohol consumption, and physical activity; and model 2 was additionally adjusted for depressive mood, cognitive impairment, activities of daily living, falls, hypertension, diabetes, and dyslipidemia. We did not adjust for PIMs because PIMs overlap a lot with medications with higher anticholinergic drug burden score. Kaplan–Meier curves were plotted to identify the cumulative incidence probabilities of all-cause mortality according to the combination of KABS scores and chronic polypharmacy with TUG test results. Additionally, we analyzed the association between the combined KABS score or chronic polypharmacy and TUG test results with the risk of all-cause mortality. Differences were considered statistically significant at *P* < 0.05.

## Results

### Baseline characteristics of study participants

Table 1 presents the baseline characteristics of 42,132 participants aged ≥ 65 years. Among them, 53.2% (*n* = 22,395) were women. The mean BMI was 24.2 ± 3.0 kg/m^2^, and the most prevalent BMI was ≥ 25 kg/m^2^. The proportions of never smokers, risky alcohol drinkers, and regular exercisers were 56.9%, 51.6%, and 54.3%, respectively. The proportions of patients with hypertension, diabetes, and dyslipidemia were 47.3%, 16.3%, and 22.1%, respectively. The mean value of TUG test results was 9.4 ± 6.5 s, and the prevalence of abnormal TUG test results was 38.4%. The proportions of patients with cognitive impairments, depressive moods, and falls were 21.6%, 50.5%, and 14.4%, respectively. The proportion of patients with normal activities of daily living was 62.5%.

**Table 1:** Baseline characteristics of participants

	Total (*n* = 42,132)
Sex (women)	22,395 (53.2)
Income (lowest quintile)	13,099 (30.9)
BMI (kg/m^2^)	24.2 ± 3.0
<18.5	1,006 (2.4)
18.5–23	13,473 (32.0)
23–25	11,598 (27.5)
≥25	16,055 (38.1)
Cigarette Smoking	
Never	18.170 (56.9)
Ex-smoker	3,856 (12.1)
Current smoker	9,913 (31.0)
At-risk alcohol drinking	12,849 (51.6)
Regular exercise	21,250 (54.3)
Hypertension	19,918 (47.3)
Diabetes mellitus	6,858 (16.3)
Dyslipidemia	9,291 (22.1)
Timed Up and Go test (s)	9.4 ± 6.5
<10	25,113 (61.6)
≥10	15,667 (38.4)
Cognitive impairment	9,007 (21.6)
Depressive mood	21,065 (50.5)
Falls	5,900 (14.4)
Normal activity of daily living	25,615 (62.5)

Values are presented as mean ± standard deviation or number (percentage)

Abbreviations: BMI, body mass index

### Association of anticholinergic burden score and chronic polypharmacy with abnormal TUG test results

Table 2 shows the association between the KABS score and chronic polypharmacy with abnormal TUG test results. The KABS scores of 0, 1–2, and ≥ 3 occurred in 38,797 (92.1%), 2,234 (5.3%), and 1,101 (2.6%) patients, respectively. The prevalence of chronic polypharmacy was 4.0%. After adjusting for confounding variables (model 3), compared with participants with KABS scores of 0, the OR for abnormal TUG test results was 1.27 times higher in those with KABS scores ≥ 3 (OR 1.27, 95% CI: 1.02–1.58). Chronic polypharmacy and abnormal TUG test results were not significantly associated.

**Table 2:** Association of anticholinergic burden score and chronic polypharmacy with abnormal Timed Up and Go test

		Total (*n* = 42,132)	Abnormal TUG (*n* = 15,667)	Crude OR (95% CI)	Model 1^a^ aOR (95% CI)	Model 2^b^ aOR (95% CI)	Model 3^c^ aOR (95% CI)
KABS score	0	38,797 (92.1)	14,250 (91.0)	1	1	1	1
	1–2	2,234 (5.3)	937 (6.0)	1.26 (1.15–1.37)	1.23 (1.13–1.35)	1.10 (0.94–1.28)	1.09 (0.93–1.27)
	≥ 3	1,101 (2.6)	480 (3.1)	1.13 (1.03–1.24)	1.33 (1.17–1.50)	1.30 (1.05–1.62)	1.27 (1.02–1.58)
	*P* for trend			< 0.001	< 0.001	0.009	0.020
Chronic polypharmacy	No	39,148 (96.0)	14,976 (95.6)	1	1	1	1
	Yes	1,632 (4.0)	691 (4.4)	1.19 (1.07–1.31)	1.18 (1.06–1.30)	1.08 (0.91–1.29)	1.05 (0.88–1.25)

KABS, Korean Anticholinergic Burden Scale; OR, odds ratio; CI, confidence interval; aOR, adjusted odds ratio; TUG, Timed Up and Go test

^a^Model 1 was adjusted for sex, body mass index, and income

^b^Model 2 was adjusted for sex, body mass index, income, smoking status, alcohol consumption, and physical activity

^c^Model 3 was adjusted for sex, body mass index, income, smoking status, alcohol consumption, physical activity, depressive mood, cognitive impairment, activities of daily living, and falls

### Association of anticholinergic burden score and chronic polypharmacy with all-cause mortality risk

Table 3 presents the HRs of all-cause mortality based on the KABS score and chronic polypharmacy. Of the total participants, 3,228 (7.66%) died during the 5.7-years mean follow-up. After adjusting for confounding variables (model 2), compared with participants with KABS score of 0, the HRs for all-cause mortality were 1.55 times higher in those with KABS scores of 1–2 (HR 1.55, 95% CI: 1.26–1.91) and 1.78 times higher in those with KABS scores ≥ 3 (1.78, 1.35–2.35) (P for trend < 0.001). The HR for all-cause mortality increased by 69% among participants with chronic polypharmacy (HR 1.69, 95% CI: 1.35–2.10), compared to among those without chronic polypharmacy.

**Table 3:** Adjusted hazard ratio of all-cause mortality according to anticholinergic burden score and chronic polypharmacy

		Number	Event	Duration (PY)	IR^a^	Crude HR (95% CI)	Model 1^b^ aHR (95% CI)	Model 2^c^ aHR (95% CI)
KABS score	0	38,797	2,812	293,562	9.6	1	1	1
	1–2	2,234	268	16,567	16.2	1.69 (1.49–1.92)	1.64 (1.34–2.01)	1.55 (1.26–1.91)
	≥ 3	1,101	148	8,051	18.4	1.93 (1.63–2.28)	1.94 (1.48–2.56)	1.78 (1.35–2.35)
	P for trend					< 0.001	< 0.001	< 0.001
Chronic polypharmacy	No	39,148	2,973	305,963	9.7	1	1	1
	Yes	1,632	255	12,217	20.9	2.17 (1.91–2.46)	2.11 (1.71–2.60)	1.69 (1.35–2.10)

KABS, Korean Anticholinergic Burden Scale; PY, person-years; IR, incidence rate; HR, hazard ratio; CI, confidence interval; aHR, adjusted hazard ratio

^a^Incidence per 1000 PY.

^b^Model 1 was adjusted for sex, body mass index, income, smoking status, alcohol consumption, and physical activity

^c^Model 2 was adjusted for sex, body mass index, income, smoking status, alcohol consumption, physical activity, depressive mood, cognitive impairment, activities of daily living, falls, hypertension, diabetes, and dyslipidemia

### Association of combined anticholinergic burden score or chronic polypharmacy and TUG test results with all-cause mortality risk

Table 4 presents the association between the combined KABS score and TUG test results or combined chronic polypharmacy and TUG test results with the risk of all-cause mortality. After adjusting for confounding variables (model 2), compared with individuals with normal TUG test results and a KABS score of 0 (reference), the HRs for all-cause mortality were 1.28 times higher in those with abnormal TUG test results and KABS scores of 0 (HR 1.28, 95% CI: 1.14–1.43), 1.38 times higher in those with normal TUG test results and KABS scores of 1–2 (HR 1.38, 95% CI: 1.03–1.83), 2.13 times higher in those with abnormal TUG test results and KABS scores of 1–2 (HR 2.13, 95% CI: 1.56–2.90), 1.63 times higher in those with normal TUG test results and KABS scores ≥ 3 (HR 1.38, 95% CI: 1.03–1.83), and 2.42 times higher in those with abnormal TUG test results and KABS scores ≥ 3 (HR 2.42, 95% CI: 1.61–3.64) (P for trend < 0.001). A similar trend was observed in the combined chronic polypharmacy and TUG test results. Compared with those with normal TUG test results without chronic polypharmacy, the HR for all-cause mortality increased by 102% among individuals with chronic polypharmacy and abnormal TUG test results (HR 2.02, 95% CI: 1.42–2.87), by 78% among those with chronic polypharmacy and normal TUG test results (HR 1.78, 95% CI: 1.34–2.37), and by 32% among those with abnormal TUG test results without chronic polypharmacy (HR 1.32, 95% CI: 1.18–1.48) (P for trend < 0.001).

**Table 4:** Adjusted HR of all-cause mortality of anticholinergic burden score and chronic polypharmacy combined with TUG test

		TUG	Number	Event	Duration (PY)	IR^a^	Crude HR (95% CI)	Model 1^b^ aHR (95% CI)	Model 2^c^ aHR (95% CI)
KABS score	0	Normal	23,311	1,553	276,219	8.8	1	1	1
		Abnormal	14,250	1,155	107,578	10.1	1.22 (1.13–1.31)	1.30 (1.16–1.46)	1.28 (1.14–1.43)
	1–2	Normal	1,220	129	9,068	12.0	1.62 (1.35–1.94)	1.41 (1.06–1.88)	1.38 (1.03–1.83)
		Abnormal	937	122	6,937	14.7	2.00 (1.66–2.41)	2.31 (1.71–3.14)	2.13 (1.56–2.90)
	≥ 3	Normal	582	70	4,248	13.0	1.87 (1.48–2.39)	1.78 (1.21–2.61)	1.63 (1.11–2.40)
		Abnormal	480	69	3,510	15.5	2.24 (1.76–2.85)	2.65 (1.77–3.98)	2.42 (1.61–3.64)
	*P* for trend						< 0.001	< 0.001	< 0.001
Chronic polypharmacy	No	Normal	24,172	1,615	182,744	8.8	1	1	1
		Abnormal	14,976	1,242	113,013	10.4	1.24 (1.15–1.34)	1.35 (1.21–1.51)	1.32 (1.18–1.48)
	Yes	Normal	941	137	6,792	17.1	2.30 (1.93–2.74)	2.26 (1.73–2.96)	1.78 (1.34–2.37)
		Abnormal	691	104	5,012	17.1	2.36 (1.94 − 2.89)	2.44 (1.73–3.44)	2.02 (1.42–2.87)
	P for trend						< 0.001	< 0.001	< 0.001

Abbreviations: KABS, Korean Anticholinergic Burden Scale; PY, person-years; IR, incidence rate; HR, hazard ratio; CI, confidence interval; aHR, adjusted hazard ratio; TUG, Timed Up and Go test

^a^Incidence per 1000 person-years

^b^Model 1 was adjusted for sex, body mass index, income, smoking status, alcohol consumption, and physical activity

^c^Model 2 was adjusted for sex, body mass index, income, smoking status, alcohol consumption, physical activity, depressive mood, cognitive impairment, activities of daily living, falls, hypertension, diabetes, and dyslipidemia

Abbreviations: TUG, Timed Up and Go

**Fig. 1:**
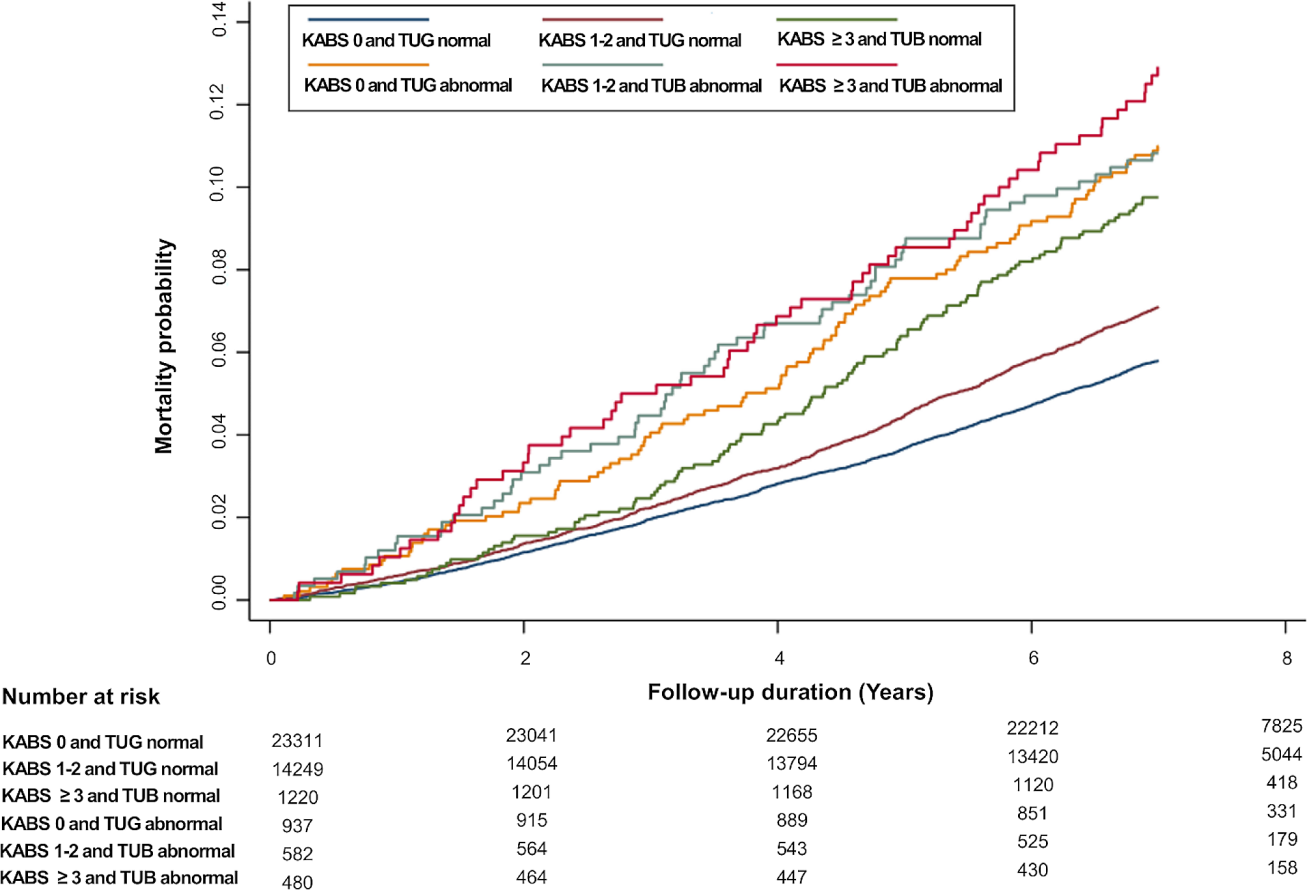
All-cause mortality based on combined anticholinergic burden scores and Timed Up and Go test. Abbreviations: KABS, Korean Anticholinergic Burden Scale; TUG, Timed Up and Go

**Fig. 2:**
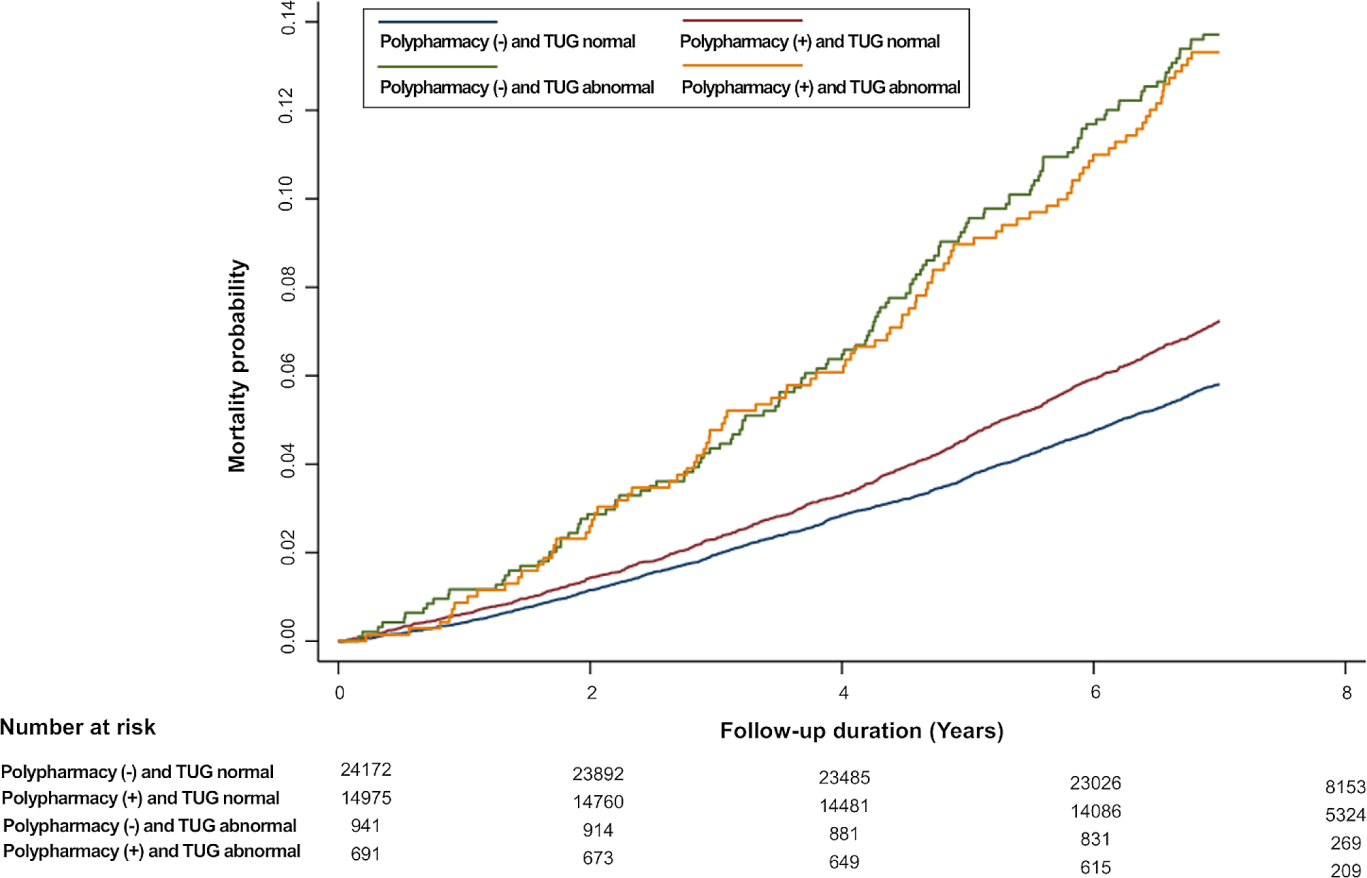
All-cause mortality based on combined chronic polypharmacy and Timed Up and Go test Abbreviations: TUG, Timed Up and Go

As shown in Fig. 1, the cumulative incidence probabilities of all-cause mortality were higher in individuals with a higher KABS score and abnormal TUG results than in those with a KABS score of 0 and normal TUG results. Figure 2 shows that the cumulative incidence probabilities of all-cause mortality increased among individuals with chronic polypharmacy and abnormal TUG test results compared to among those without chronic polypharmacy.

## Discussion

The present study showed the association of anticholinergic drug burden and chronic polypharmacy with functional decline and all-cause mortality among older people based on a large-scale national cohort study. As anticholinergic burden increased, the association between functional decline and risk of all-cause mortality increased. Chronic polypharmacy also increased the risk of all-cause mortality. Furthermore, the combination of a higher anticholinergic drug burden score or chronic polypharmacy and abnormal TUG test results increased the risk of all-cause mortality. Therefore, our results suggest that anticholinergic drug burden and not chronic polypharmacy was association with functional decline, and anticholinergic drug burden and chronic polypharmacy may be independent risk factors for all-cause mortality in older people.

Several previous studies have shown that anticholinergic drug burden and chronic polypharmacy are associated with frailty in older people. One study including 17,084 community-dwelling adults aged ≥ 65 years demonstrated that the OR of frailty was 4.78 times higher in individuals with current anticholinergic medications (OR 4.78, 95% CI: 4.30–5.31) compared to in those without anticholinergic medications [19]. A study of 3,058 community-dwelling German adults aged 57–84 years showed that OR of frailty was 2.30 times higher in participants taking 5–9 medications (OR 2.30, 95% CI: 1.60–3.31) and 4.97 times higher in those taking ≥ 10 medications (OR 4.97, 95% CI: 2.97–8.32) after adjusting for confounding variables compared to in those taking 0–4 medications [20]. A previous study of 4,402 North Americans showed that taking a greater number of medications was associated with an increased risk of incident frailty compared to taking 0–3 medications (HR [95% CI]; 1.55 [1.22–1.96] for 4–6 medications, 2.47 [1.78–3.43] for ≥ 7 medications) [12]. Further, a study of 785 patients aged ≥ 75 years with blood cancers reported that the OR for frailty was 2.82 times higher in older people with polypharmacy (≥ 8 medications) compared to in those without polypharmacy (OR 2.82, 95% CI: 1.92–4.17) [21]. In addition, as the anticholinergic risk score increased by one point, the OR for frailty increased by 19% (OR 1.19, 95% CI: 1.03–1.39) [21]. However, unlike this previous finding, our study found that polypharmacy based on taking five medications was not associated with frailty in older people. The difference from previous studies is possibly due to exclusion of non-chronic medications which is taken by participants shorten than 250 days.

Anticholinergic drug burden and polypharmacy have been reported to increase the risk of all-cause mortality [22–25]. A previous study, which included 807 patients aged ≥ 65 years who were followed up for 12 months after discharge reported that the HR for 1-year all-cause mortality was 1.69 times higher in patients with an anticholinergic drug burden ≥ 2 (HR 1.69, 95% CI: 1.09–2.65) compared to in those with an anticholinergic drug burden of 0 [22]. A previous retrospective study of 1,497 older people and anticholinergic drug burden defined using the modified-Anticholinergic and Drug Scale showed that the HR for all-cause mortality increased by 9% among participants with anticholinergic burden (HR 1.09, 95% CI: 1.04–1.15) compared to among those without anticholinergic burden [23]. One study involving South Korean older people reported that polypharmacy was associated with an increased risk of all-cause mortality (HR 1.25, 95% CI: 1.24–1.25) [24]. A nationally representative study showed that compared to the HRs of individuals aged ≥ 50 years taking no medications, the HRs for all-cause mortality increased by 51% among those taking 5–9 medications (HR 1.51, 95% CI: 1.05–1.21) and by 129% among those taking ≥ 10 medications (HR 2.29, 95% CI: 1.40–3.75) [25]. Additionally, we found that the risk of all-cause mortality increased when anticholinergic drug burden or polypharmacy was combined with frailty. Therefore, our study provides further evidence that anticholinergic drug burden and polypharmacy increase the risk of all-cause mortality in frail older people.

Our study showed the difference in the associations of anticholinergic drug burden and chronic polypharmacy with function decline and all-cause mortality. One study including 318 hospitalized patients aged ≥ 65 years presented that anticholinergic drug burden had a higher association with in-hospital mortality than polypharmacy [26]. However, the subjects of this previous study were a small number of inpatients and outcome of study was in-hospital mortality, which were different from our study including a large nationwide cohort. Additionally, while other studies on polypharmacy defined the number of medications, our study used chronic polypharmacy, in which medications intake was used continuously over a certain period of time.

Anticholinergic drug burden and polypharmacy may induce frailty and all-cause mortality. First, anticholinergic drug burden and polypharmacy contribute to comorbidities or factors, including pre-frailty conditions such as weight loss, balance disorders, and nutritional deficiencies [27]. Second, these conditions could lead to poor clinical outcomes such as unplanned hospitalizations [28] and adverse drug effects [29]. In particular, adverse drug reactions can trigger a prescription cascade, which can increase the number of medications and make patients more vulnerable to adverse effects [30].

The present study had some limitations. First, the variability of the TUG test results could not be confirmed because the test was performed at a single health check-up. Second, the present study avoided causality-related conclusions because of its observational design. Third, because we defined anticholinergic drug burden and chronic polypharmacy based on the claims database, it is possible that they were underestimated. Additionally, each drug of anticholinergic burden could not be known because of access restrictions of database. Fourth, the health status and functional capacity of older adults, such as their ability to walk, should be considered when interpreting our results because they can participate in health checkups. In addition, because of study design as a retrospective cohort, there could be some confounding factors that could not be fully addressed. Finally, although many confounding variables were considered, there were potential confounding variables that were not included in the NHIS-Senior database. In addition, although physical activity, anthropometric measurements, and metabolic diseases were included, other conditions like osteoporosis were not included because they were difficult to accurately define using the diagnostic code, medication, and imaging test. The present study included comorbidities that were highly associated with frailty and sarcopenia and were clearly definable. Nevertheless, the present study presents the association of anticholinergic drug burden and chronic polypharmacy with the risk of function decline and all-cause mortality among older people using a nationwide representative cohort of South Koreans. In addition, our study is the first to show that the combination of a higher anticholinergic drug burden or chronic polypharmacy score and abnormal TUG test results increased the risk of all-cause mortality among older people.

## Conclusion

In conclusion, the present study showed that Anticholinergic drug burden shows a better association with functional decline than chronic polypharmacy, and the use of medications and functional decline may be important risk factors for all-cause mortality among South Korean older people. In addition, the risk of all-cause mortality was further increased in older people with combined higher anticholinergic drug burden scores or chronic polypharmacy and function decline. Therefore, clinicians need to be careful about the use of medications and functional decline among older people.

### Electronic supplementary material

Below is the link to the electronic supplementary material.

Supplementary Table S1: Medication lists according to the Korean Anticholinergic Burden Scale

## Data Availability

This study was performed using the Korean National Health Insurance System database, and the results do not necessarily represent the opinion of the National Health Insurance Corporation. Restrictions apply to the availability of these data, which were used under license for this study, and so are not publicly available because of ethical issues and data protections. Data are available from the Korea National Health Insurance Sharing Service Institutional Data Access/Ethics Committee (https://nhiss.nhis.or.kr/bd/ay/ bdaya001iv.do) for researchers who meet the criteria for the access to confidential data.

## Abbreviations

aHR: Adjusted hazards ratio
aOR: Adjusted odds ratio
BMI: Body mass index
CI: Confidence interval
HR: Hazards ratio
ICD-10: International Classification of Diseases, 10th Revision
IR: Incidence rate
KABS: Korean Anticholinergic Burden Scale
NHIS-Senior: National Health Insurance Service-Senior
OR: Odds ratio
PIMS: Potentially inappropriate medications
PY: Person-years
TUG: Timed Up and Go
US: United States
