# Supplementary material for: Comparison of anticholinergic burden with chronic polypharmacy on functional decline and mortality in Korean older people: a retrospective nationwide cohort study
Source: BMC Geriatr. 2024 Jan 23;24:90. doi: 10.1186/s12877-024-04692-0 (PMC10807110; doi:10.1186/s12877-024-04692-0)
Supplement: Supplementary file 1 — Supplementary Table S1: Medication lists according to the Korean Anticholinergic Burden Scale [file 12877_2024_4692_MOESM1_ESM.docx]

Supplementary Table S1 Medication lists according to the Korean Anticholinergic Burden Scale

|  | Score 3 | Score 2 | Score 1 | Score 0 |
| --- | --- | --- | --- | --- |
| Analgesics |  | pethidine  tramadol | fentanyl  codeine  hydrocodone  morphine, oxycodone | acetaminophen, aspirin, buprenorphine, celecoxib, diclofenac, etodolac, ibuprofen, ketoprofen, ketorolac, meloxicam, nabumetone, naproxen, naratriptan, piroxicam, sulindac, sumatriptan, zolmitriptan |
| Anti-dementia drug |  |  |  | donepezil, galantamine, ginkgo, memantine, rivastigmine |
| Antidepressants | amitriptyline  amoxapine  clomipramine  doxepin  imipramine | paroxetine | Bupropion, citalopram  desvenlafaxine  escitalopram  fluoxetine  fluvoxamine  mirtazapine  trazodone  venlafaxine | duloxetine  moclobemide  sertraline  tianeptine |
| Antiepileptic drugs |  | oxcarbazepine | carbamazepine | gabapentin, lamotrigine, levetiracetam, phenytoin, phenobarbital, primidone, topiramate, valproate, valproic acid |
| Antihistamine | chlorpheniramine  clemastine  hydroxyzine  mequitazine | triprolidine | levocetirizine  loratadine |  |
| Anti-infectives |  |  |  | acyclovir, amoxicillin, ampicillin, azithromycin  cefaclor, cefamandole, cefazolin, cefixime, cefoxitin, ceftibuten, ceftriaxone, cefuroxime, cephalexin, ciprofloxacin, clarithromycin, clindamycin, cycloserine, doxycycline, erythromycin, ethambutol, famciclovir, fluconazole, gentamicin, imipenem, isoniazid, levofloxacin, metronidazole, minocycline, moxifloxacin, norfloxacin, neomycin, ofloxacin, penicillin, piperacillin, pyrazinamide, rifampicin, roxithromycin, trimethoprim, terbinafine, tetracycline, vancomycin |
| Antineoplastic and  immune-modulating agents | immune-modulating agents |  |  | anagrelide, anastrozole, bicalutamide, cyclophosphamide, cyclosporine, filgrastim, 5-fluorouracil, goserelin, hydroxyurea, leuprolide, methotrexate, tamoxifen |
| Antiparkinson drugs |  | amantadine |  | entacapone, levodopa, pramipexole, ropinirole, selegiline |
| Antipsychotics | chlorpromazine  clozapine  olanzapine | levomepromazine  perphenazine  pimozide  quetiapine | amisulpride  aripiprazole  blonanserin  haloperidol  paliperidone  risperidone  ziprasidone | lithium |
| Antithrombotic agent | |  |  | clopidogrel, enoxaparin, heparin, ticlopidine, warfarin |
| Antivertigo | dimenhydrinate |  |  | betahistine |
| Anxiolytics, hypnotics, and sedatives |  |  | alprazolam  chlordiazepoxide  clonazepam  diazepam  flunitrazepam  flurazepam  lorazepam  midazolam  triazolam | buspirone  chloral  clobazam  zolpidem |
| Cardiovascular drugs |  |  | digoxin  furosemide  hydralazine | adenosine, amiloride, amiodarone, amlodipine  atenolol, atorvastatin, betaxolol, bisoprolol  candesartan, captopril, carvedilol, chlorthalidone  cholestyramine, cilostazol, clonidine, diltiazem  dobutamine, dopamine, doxazosin, enalapril  epinephrine, ezetimibe, felodipine, fenofibrate  flecainide, fluvastatin, gemfibrozil, hydrochlorothiazide, indapamide, irbesartan, isosorbide, labetalol, lercanidipine, lisinopril, losartan, lovastatin, mannitol, metoprolol  midodrine, nifedipine, nisoldipine, nitroglycerin  norepinephrine, olmesartan, omega-3-acid ethyl esters, pentoxifylline, perindopril, pravastatin, propafenone, propranolol, ramipril, rosuvastatin, simvastatin, sotalol, spironolactone, telmisartan, terazosin, timolol, ubidecarenone, valsartan, verapamil |
| Diabetes drugs |  |  |  | acarbose, glibenclamide, gliclazide, glimepiride, glipizide, insulin, metformin, nateglinide, pioglitazone, repaglinide |
| Gastrointestinal disorders | atropine  cimetropium  octylonium | cimetidine  clidinium  glycopyrrolate | loperamide  ranitidine  trimebutine | aluminum oxide, alverine, betaine, bisacodyl  d-sorbitol, domperidone, esomeprazole  famotidine, fenoverine, lactic acid, lactobacillus casei, lactulose, lansoprazole, magnesium  mesalazine, metoclopramide, misoprostol  nizatidine, omeprazole, pantoprazole, papaverine  polycarbophil, psyllium, rabeprazole, simethicone  sucralfate hydrate, sulfasalazine, tiropramide |
| Genitourinary system and sex hormone | flavoxate  imidafenacin  oxybutynin propiverine  solifenacin  tolterodine, trospium |  |  | bethanechol, conjugated estrogens, danazol  estradiol, finasteride, medroxyprogesterone  megestrol, progesterone, sildenafil, tamsulosin  testosterone, tibolone |
| Hormonal preparations |  |  | hydrocortisone  prednisolone | carbimazole, desmopressin, dexamethasone  fludrocortisone, glucagon, levothyroxine, triamcinolone methylprednisolone, octreotide, propylthiouracil |
| Minerals and vitamins |  |  |  | ascorbic acid, calcitriol, folic acid, pyridoxine, thiamine |
| Musculoskeletal agents | orphenadrine  pridinol | cyclobenzaprine  tizanidine | baclofen  methocarbamol | alendronate, allopurinol, chondroitin, colchicine  dantrolene, eperisone, etidronate, pamidronate  raloxifene, risedronate, vecuronium |
| Respiratory drugs |  | cloperastine | guaifenesin | benzonatate, bromhexine, montelukast, acetylcysteine, phenylephrine, pseudoephedrine, salbutamol |
| Miscellaneous |  |  |  | acetazolamide, acitretin, acetyl l-carnitine, l-carnitine, darbepoetin α, deferasirox, flumazenil, glucose, hydroxychloroquine, iron, lidocaine, methylphenidate, modafinil, naloxone, pilocarpine, protamine, pyridostigmine  sodium, tuberculin |
